# Genetically proxied impaired GIPR signaling and risk of 6 cancers

**DOI:** 10.1016/j.isci.2023.106848

**Published:** 2023-05-09

**Authors:** Miranda Rogers, Dipender Gill, Emma Ahlqvist, Tim Robinson, Daniela Mariosa, Mattias Johansson, Ricardo Cortez Cardoso Penha, Laure Dossus, Marc J. Gunter, Victor Moreno, George Davey Smith, Richard M. Martin, James Yarmolinsky

**Affiliations:** 1 MRC Integrative Epidemiology Unit, University of Bristol, BS8 2BN Bristol, UK; 2 Population Health Sciences, Bristol Medical School, University of Bristol, BS8 2PS Bristol, UK; 3 Department of Epidemiology and Biostatistics, School of Public Health, Imperial College London, W2 1PG London, UK; 4 Chief Scientific Office, Research and Early Development, Novo Nordisk, 2300 Copenhagen, Denmark; 5 Department of Clinical Sciences, Lund University, Lund, 22362 Malmö, Sweden; 6 Genomic Epidemiology Branch, International Agency for Research on Cancer (IARC/WHO), 69007 Lyon, France; 7 Nutrition and Metabolism Branch, International Agency for Research on Cancer (IARC/WHO), 69007 Lyon, France; 8 Biomarkers and Susceptibility Unit, Oncology Data Analytics Program, Catalan Institute of Oncology (ICO), 08908 L'Hospitalet de Llobregat, Barcelona, Spain; 9 Colorectal Cancer Group, ONCOBELL Program, Bellvitge Biomedical Research Institute(IDIBELL), 08908 L'Hospitalet de Llobregat, Barcelona, Spain; 10 Consortium for Biomedical Research in Epidemiology and Public Health (CIBERESP), 28029 Madrid, Spain; 11 Department of Clinical Sciences, Faculty of Medicine, University of Barcelona, 08036 Barcelona, Spain; 12 University Hospitals Bristol and Weston NHS Foundation Trust, National Institute for Health Research Bristol Biomedical Research Centre, University of Bristol, BS8 2BN Bristol, UK

**Keywords:** Health sciences, Genetics, Cancer

## Abstract

Preclinical and genetic studies suggest that impaired glucose-dependent insulinotropic polypeptide receptor (GIPR) signaling worsens glycemic control. The relationship between GIPR signaling and the risk of cancers influenced by impaired glucose homeostasis is unclear. We examined the association of a variant in *GIPR*, rs1800437 (E354Q), shown to impair long-term GIPR signaling and lower circulating glucose-dependent insulinotropic peptide concentrations, with risk of 6 cancers influenced by impaired glucose homeostasis (breast, colorectal, endometrial, lung, pancreatic, and renal) in up to 235,698 cases and 333,932 controls. Each copy of E354Q was associated with a higher risk of overall and luminal A-like breast cancer and this association was consistent in replication and colocalization analyses. E354Q was also associated with higher postprandial glucose concentrations but diminished insulin secretion and lower testosterone concentrations. Our human genetics analysis suggests an adverse effect of the *GIPR* E354Q variant on breast cancer risk, supporting further evaluation of GIPR signaling in breast cancer prevention.

## Highlights

• GIPR signaling may influence cancer risk
• We tested the effect of a functional variant in *GIPR* (E354Q) on risk of 6 cancers
• E354Q was associated with increased risk of overall and luminal A-like breast cancer
• Further evaluation of GIPR signaling in breast cancer prevention is warranted

## Graphical abstract

**Figure:**
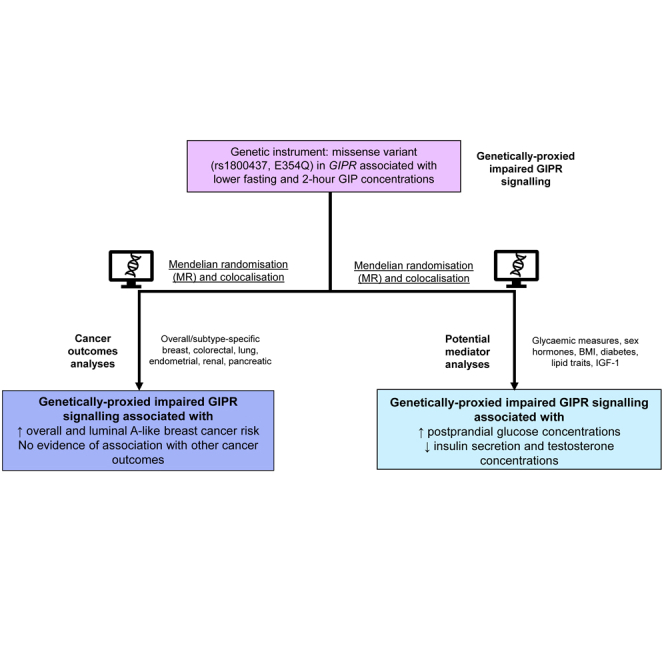

## Introduction

Preclinical and epidemiological studies suggest an important role of dysregulated metabolism in cancer development, in particular carcinogenic effects of sustained elevated insulin levels.^1^^,^^2^ Hyperinsulinaemia has consistently been associated with risk of several cancers in both observational and genetic epidemiological studies.^3^^,^^4^^,^^5^^,^^6^^,^^7^^,^^8^^,^^9^ *In vitro* studies have demonstrated that insulin signaling is mitogenic on cancer cells and can induce cell migration, providing possible mechanisms for carcinogenesis.^10^ Enhanced understanding of molecular mechanisms regulating insulin signaling could inform the development of potential therapeutic strategies for cancer prevention.

Glucose-dependent insulinotropic peptide (GIP) is one of two incretin hormones, along with glucagon-like peptide-1 (GLP1), that are produced in response to nutrient consumption, maintaining glucose homeostasis by increasing insulin and lowering glucagon secretion.^11^ In a phase 3 clinical trial, tirzepatide, a dual GIPR/GLP1R agonist, was shown to confer superior HbA_1c_ control as compared to GLP1R agonism alone and has recently been approved by the U.S. Food and Drug Administration (FDA) for type 2 diabetes treatment.^12^^,^^13^ By potentiating postprandial insulin secretion and increasing blood insulin levels, there is some concern that pharmacological agonism of the GIPR signaling pathway could increase risk of hyperinsulinemia-driven cancers.^14^ GIPR signaling has also been previously implicated in bone growth and cardiovascular disease. A *GIPR* missense variant rs1800437 (E354Q, C allele), indexing long-term reduced GIPR signaling, has been shown to be associated with increased bone mineral density and increased risk of fractures.^15^ Higher fasting GIP levels mediated via this variant have been linked to an increased risk of coronary artery disease (CAD) and myocardial infarction, though subsequent analyses suggested that fasting GIP and CAD associations are likely to be driven through distinct genetic signals at this locus.^16^^,^^17^ In addition, fasting plasma GIP levels have been linked to an increased mean common carotid artery intima-media thickness and increased GIP levels following an oral glucose tolerance test have been associated with long-QT syndrome type 2 and an unhealthy fat distribution.^18^^,^^19^^,^^20^ The few epidemiological studies that have examined the relationship between circulating GIP concentrations and cancer risk have generated conflicting results.^21^^,^^22^^,^^23^ Naturally occurring variation in genes encoding drug targets can be leveraged to predict the effect of pharmacological perturbation of these targets on disease risk (“drug-target Mendelian randomization [MR]”).^24^ Since germline genetic variants are randomly assorted at meiosis and fixed at conception, such studies should be less prone to confounding than conventional observational studies and cannot be influenced by reverse causation.^25^^,^^26^ In addition, drug-target MR permits the effect of the long-term perturbation of drug targets on cancer risk to be examined. This is advantageous when evaluating cancer outcomes given long induction periods for cancer development and the number of emerging drugs that do not have long-term efficacy data.^26^^,^^27^

Here, we used a missense variant in *GIPR,* previously shown to result in impaired long-term GIPR signaling and decreased fasting and 2-h GIP concentrations, to predict the potential effect of such impaired GIPR signaling on the risk of 6 cancers influenced by hyperinsulinemia (overall and histotype-specific breast, colorectal, endometrial, lung, pancreatic, and renal cancers).^28^^,^^29^ We tested findings for replication in the Finngen Consortium and employed colocalization to evaluate their robustness to violations of MR assumptions. Finally, we used this variant to examine potential downstream mediators of GIPR signaling (i.e. various measures of childhood and adult adiposity, fasting and postprandial glucose and insulin, other glycemic traits, endogenous sex hormones, and lipids), to identify possible mechanisms underpinning the effect of impaired GIPR signaling on cancer risk.

## Results

Characteristics of genetic variants used to proxy all traits are presented in Table S1. F-statistics for genetic instruments for these traits ranged from 57.7 to 30,028.7, suggesting that our analyses were unlikely to suffer from weak instrument bias (Table 1).

**Table 1:** Instrument strength estimates across all traits examined

Trait (units)	N of SNPs	R^2^	F-stats
Bioavailable testosterone (ln-transformed, nmol/L)	178	0.054	10,760.2
Total testosterone (inverse normal rank transformed, nmol/L)	256	0.074	18,454.2
Type 2 diabetes (BMI adj.)	58	0.017	5,241.8
2 h glucose (mmol L^−1^)	14	0.0028	790.3
HbA_1c_ (%)	64	0.026	7,552.5
BMI (sex-combined) (SD)	419	0.061	30,028.7
BMI (female) (SD)	36	0.014	2,463.7
Comparative body size at age 10	209	0.035	16,720.0

R^2^ is an estimate of the proportion of variance in each trait explained by the instrument. An F-statistic >10 is conventionally used to indicate that instruments are unlikely to suffer from weak instrument bias.^30^ In analyses of the effect of E354Q on breast cancer risk scaled to the effect of this variant on GIP concentrations, r^2^ and F-statistics for fasting and 2-h GIP concentrations were: 0.0073 and 57.7, 0.0085 and 64.0, respectively. Summary genetic association data on fasting and 2-h GIP concentrations from Almgren et al. were obtained from the MDC subcohort because of denser variant coverage as compared to the PPP-Botnia study. HbA_1c_ = glycated hemoglobin, BMI = body mass index (adult), comparative body size at age 10 = recall of an individual’s body size at age 10 as compared to average.

### Association of E354Q with cancer risk

**Figure 1:**
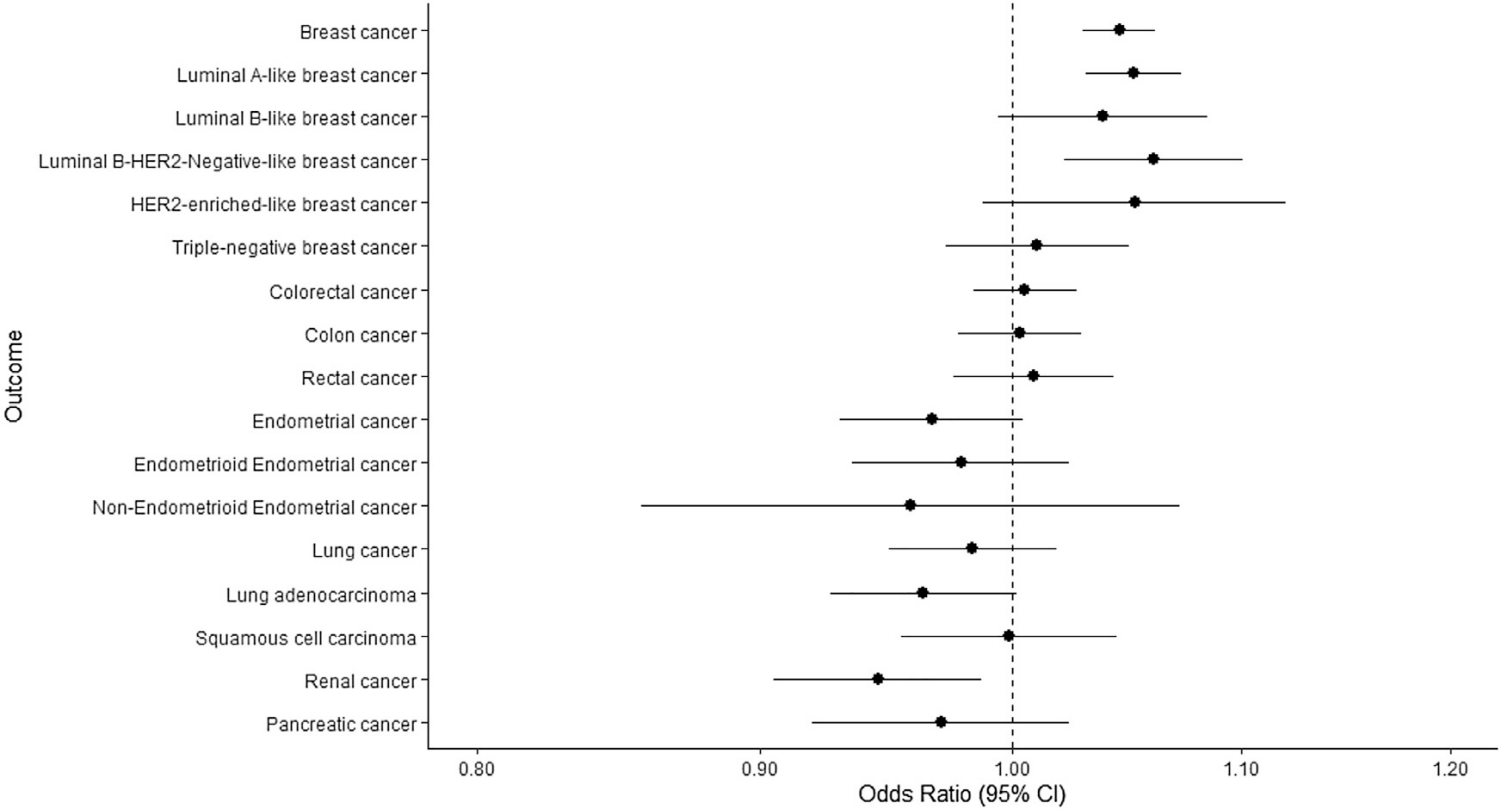
Association between E354Q and overall and histotype-specific breast, endometrial, colorectal, lung, renal, and pancreatic cancer risk Odds ratio represents the exponential increase in odds per copy of E354Q (rs1800437, C allele).

Each copy of E354Q was strongly associated with a higher risk of breast cancer (OR:1.05, 95% confidence interval [CI]:1.03–1.06, p = 6.26x10^−9^)(Figure 1, Table S2). In histological subtype-stratified analyses, E354Q was also strongly associated with a higher risk of luminal A-like (OR:1.05, 95% CI:1.03–1.07, p = 6.02x10^−7^) and luminal B HER2 negative-like breast cancer (OR:1.06, 95% CI:1.02–1.10, p = 1.82x10^−3^)(Figure 1, Table S2). When scaled to a 1 unit lowering of ln-fasting GIP concentrations mediated by this variant this represents ORs (95% CIs) of 1.80 (1.48–2.19), 1.94 (1.50–2.52), and 2.17 (1.33–3.54) for overall, luminal A-like, and luminal B HER2 negative-like breast cancer, respectively. Colocalization analysis suggested that fasting and 2-h GIP concentrations had a >99.9% posterior probability of sharing a causal variant with both overall and luminal A-like breast cancer risk within the *GIPR* locus and a >51.8% probability of sharing a causal variant with luminal B HER2 negative-like breast cancer (Table 2).

**Table 2:** Colocalization analysis results for fasting and 2-h GIP concentrations and cancer risk in the *GIPR* locus

Exposure	Outcome	H_0_	H_1_	H_2_	H_3_	H_4_
Fasting GIP	Overall breast cancer	1.84 x10^−6^	1.19 x10^−4^	3.21 x10^−4^	1.07 x10^−3^	0.999
Fasting GIP	Luminal A	1.36 x10^−4^	8.76 x10^−4^	3.24 x10^−4^	1.09 x10^−3^	0.998
Fasting GIP	Luminal B HER2 Negative	6.43 x10^−2^	0.42	4.10 x10^−4^	2.13 x10^−3^	0.52
Fasting GIP	Renal cancer	0.11	0.70	1.02 x10^−3^	6.36 x10^−3^	0.18
2-h GIP	Overall breast cancer	9.14 x10^−7^	1.25 x10^−5^	1.59 x10^−4^	1.18 x10^−3^	0.999
2-h GIP	Luminal A	6.68 x10^−5^	9.14 x10^−4^	1.59 x10^−4^	1.18 x10^−3^	0.998
2-h GIP	Luminal B HER2 Negative	0.032	0.46	9.68 x10^−14^	2.23 x10^−3^	0.53
2-h GIP	Renal cancer	0.053	0.73	4.95 x10^−4^	6.56 x10^−3^	0.21

H_0_-H_4_: posterior probabilities of the associations between the 2 traits examined, evaluating 5 different configurations.

H_0_: Neither trait has an association in the region.

H_1_: The first trait has an association in the region but the second does not.

H_2_: The second trait has an association in the region but the first does not.

H3: Both traits have an association in the region but have different causal variants.

H4: Both traits have an association in the region and share the same causal variant.

In analyses across five other cancer sites, there was weak evidence for an association of E354Q with a lower risk of renal cancer (OR:0.95, 95% CI:0.91–0.99, p = 0.01), but little evidence of association of this variant with risk of 5 other cancers examined (Figure 1, Table S2). In colocalization analysis, there was little evidence to support one or more shared causal variants for fasting or 2-h GIP concentrations and renal cancer risk in *GIPR* (H_4_<21.2%; Table 2).

### Replication analyses in FinnGen and exploratory analyses in *BRCA1/2* mutation carriers

Findings for an association of E354Q with breast cancer risk were replicated in an independent sample of 8,401 cases and 99,321 controls in the FinnGen consortium (OR:1.06, 95% CI:1.02–1.09, p = 1.09x10^−3^). In exploratory analyses in *BRCA1* or *BRCA2* mutation carriers, there was little evidence of association of E354Q with breast cancer risk (*BRCA1*:OR 1.00, 95% CI:0.96–1.05, p = 0.98; *BRCA2*:OR:1.04, 95% CI:0.98–1.11, p = 0.16).

### Type 2 diabetes, body mass index, glycemic traits, lipids, and sex hormones as potential mediators of an association of E354Q with breast cancer risk

**Figure 2:**
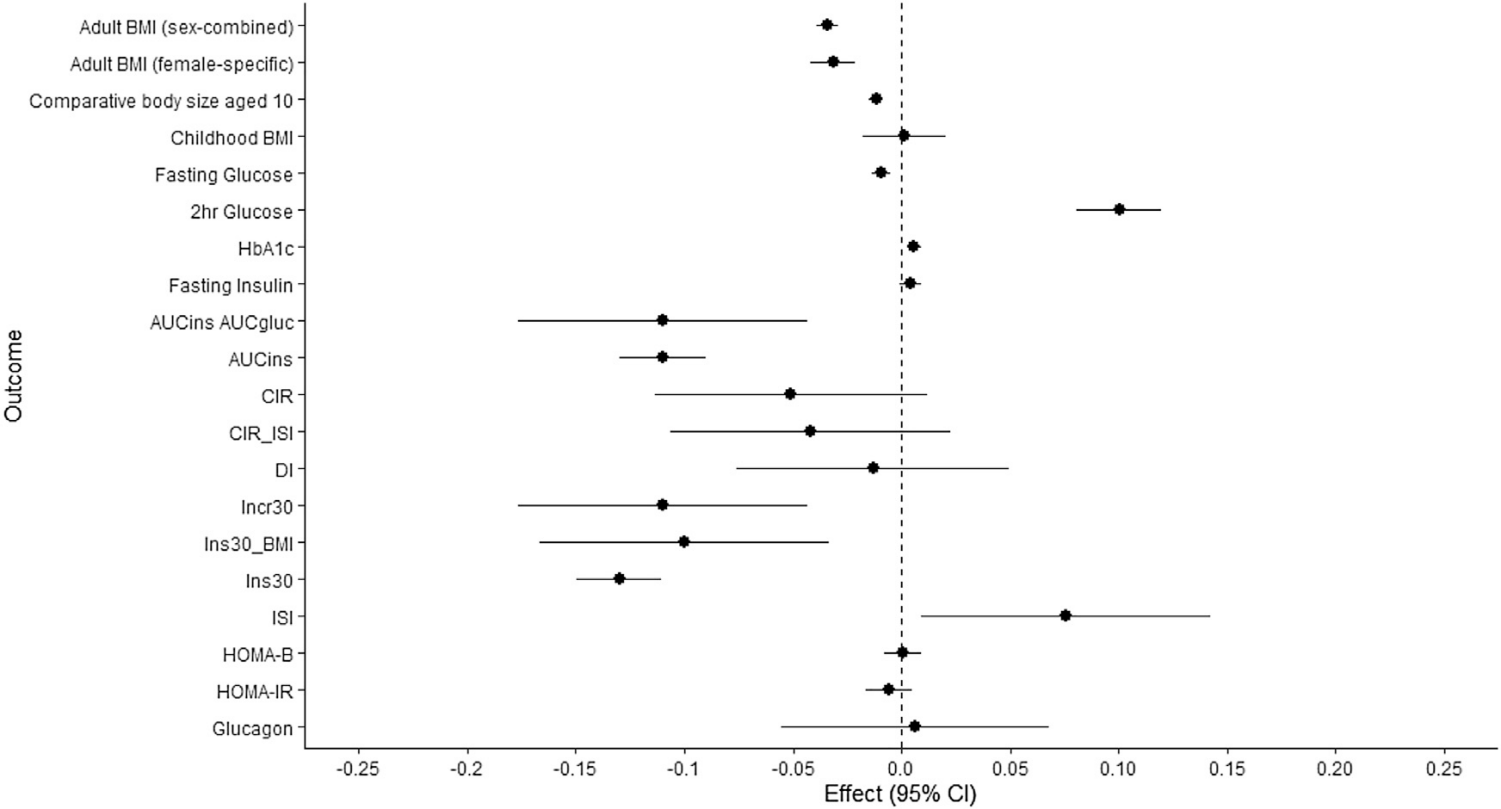
Association between E354Q and glycemic traits and adiposity measures Effect represents the change in continuous trait per copy of E354Q (rs1800437, C allele). HbA1c = glycated hemoglobin, CIR = Corrected Insulin Response, calculated using 100× insulin at 30 min)/(glucose at 30 min×(glucose at 30 min–3.89); AUC_Ins_/AUC_Gluc_ (mU/mmol) = ratio of the area under the curve (AUC) for AUC insulin/AUC glucose calculated using the trapezium rule; ISI = Insulin sensitivity index, calculated using 10,000/√ (fasting plasma glucose (mg/dL)×fasting insulin×mean glucose during oral glucose tolerance test (OGTT) (mg/dL)×mean insulin during OGTT); CIR_ISI = CIR adjusted for insulin sensitivity index; DI = disposition index, calculated using CIR×ISI; Ins_30_ = insulin at 30 min; Incr_30_ = incremental insulin at 30 min, calculated by insulin 30 min – fasting insulin; Ins_30_ (BMI adj.) = insulin response to glucose during the first 30 min adjusted for BMI, calculated using insulin at 30 min/(glucose at 30 min×BMI); AUCIns (mU∗min/l) = area under the curve (AUC) of insulin levels during OGTT, HOMA-IR = Homeostatic model assessment of insulin resistance, HOMA-B = Homeostatic model assessment of beta-cell function, BMI = body mass index (adult), childhood BMI = BMI in children aged between 2 and 10 years old, comparative body size at age 10 = Recall of an individual’s body size at age 10 as compared to average. Glucagon levels were from random plasma sample. Unit change in each outcome measure is as follows: adult BMI (SD), comparative body size (change from lowest to middle or middle to highest level of self-reported comparative body size), childhood BMI (SD), fasting glucose (mmol/L), 2-h glucose (mmol/L),HbA_1c_ (%), fasting insulin (natural log-transformed pmol/L), AUC_ins_/AUC_gluc_ (mU/mmol), AUC_ins_ (mU∗min/l), CIR (no units), CIR_ISI (no units), DI (no units), Incr_30_ (no units), Ins30_BMI (no units), Ins30 (no units), ISI (no units), HOMA-B (no units), HOMA-IR (no units), Glucagon (inverse-rank normalised).

**Figure 3:**
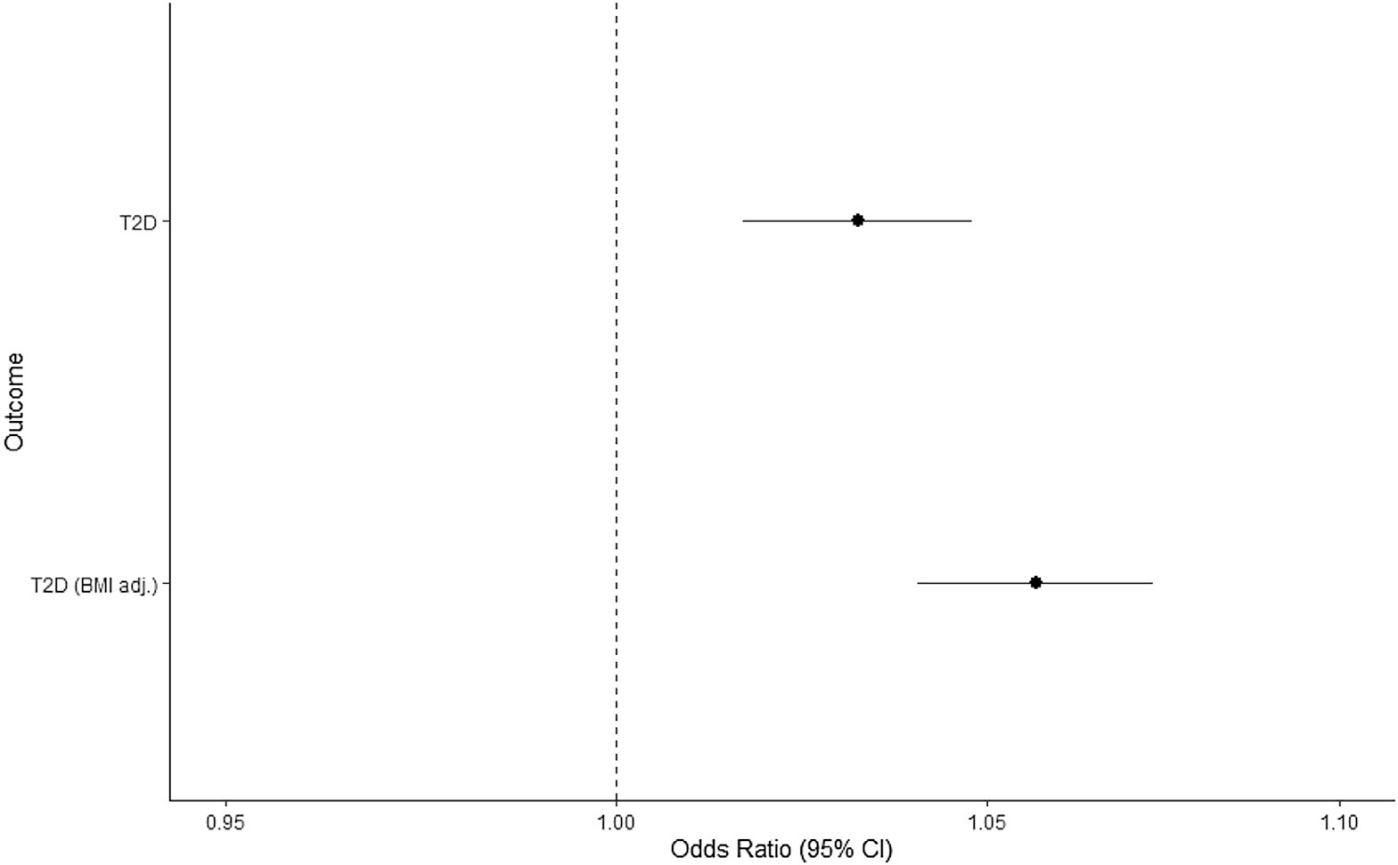
Association between E354Q and type 2 diabetes (adjusted and unadjusted for BMI) OR represents the exponential increase in odds per copy of E354Q (rs1800437, C allele).T2D = type 2 diabetes, BMI adj. = adjusted for body mass index (adult).

In combined MR and colocalization analyses, we found consistent evidence to implicate E354Q in a higher risk of type 2 diabetes (BMI adj.)(OR:1.06, 95% CI:1.04–1.07, p = 6.80x10^−12^; fasting GIP colocalization H_4_ ≥ 90.0%) and lower adult BMI (−0.034SD change, 95% CI:-0.039,-0.029, p = 7.08x10^−42^, H_4_ = 99.9%)(Figures 2, 3). The association of E354Q with BMI was consistent in sensitivity analyses using female-specific BMI association estimates (−0.032SD change, 95% CI:-0.042,-0.022, p = 5.79x10^−42^, H_4_ = 99.8%) (Figure 2, Table S3). We also found consistent evidence to implicate E354Q in smaller comparative body size aged 10 (−0.012SD change, 95% CI:-0.015,-0.0083, p = 3.10x10^11^, H_4_ = 99.9%), although there was no evidence for an association with measured BMI in children aged 2–10 (0.0014SD change, 95% CI:-0.018,0.021, p = 0.89) (Figure 2, Table S3).

Each copy of E354Q was also associated with higher 2-h glucose concentrations (0.10, 95% CI:0.08–0.12, p = 3.58x10^−24^, H_4_ = 99.9%) and lower levels of 3 measures of insulin secretion: AUC_ins_ (−0.11, 95% CI:-0.13,-0.09, p = 1.18x10^−3^, H_4_ ≥ 70.1%), AUC_ins_/AUC_gluc_ (−0.11, 95% CI:-0.17,-0.04, p = 9.85x10^−4^, H_4_ ≥ 67.6%), and Ins_30_ (−0.13, 95% CI:-0.15,-0.11, p = 1.96x10^−4^, H_4_ ≥ 80.8%) (Figure 2, Tables 3 and S3). Evidence of an association of E354Q with HbA_1c_ (0.0057% change, 95% CI:0.0026,0.0088, p = 3.67x10^−4^) and Ins_30_ (BMI adj.)(-0.10, 95% CI:-0.17,-0.03, p = 2.15x10^−3^) was also supported in colocalization analysis for 2-h, but not fasting, GIP concentrations (H_4_ = 64.2% and 52.4% for HbA_1c_ and Ins_30_ (BMI adj.), respectively)(Figure 2, Tables 3 and S3).

**Table 3:** Colocalization analysis results for fasting and 2-h GIP concentrations, BMI (sex-combined and female-specific), type 2 diabetes, and glycemic traits in the *GIPR* locus

Exposure	Outcome	H_0_	H_1_	H_2_	H_3_	H_4_
Fasting GIP	Fasting glucose	9.17 x 10^−4^	5.91 x 10^−3^	0.11	0.69	0.20
	2h glucose	2.21 x 10^−21^	1.42 x 10^−20^	3.18 x 10^−4^	1.05 x 10^−3^	0.999
	HbA_1c_	0.027	0.17	0.047	0.30	0.45
	AUC_ins_/AUC_gluc_	0.040	0.26	3.68 x 10^−3^	0.023	0.68
	AUC_ins_	0.039	0.25	8.69 x 10^−4^	4.91 x 10^−3^	0.70
	Incr_30_	0.011	0.068	0.096	0.61	0.20
	Ins_30_ (BMI adj.)	0.061	0.40	7.65 x 10^−3^	0.049	0.48
	Ins_30_	5.85 x 10^−3^	0.038	2.01 x 10^−2^	0.13	0.81
	ISI	0.11	0.74	3.61 x 10^−4^	2.18 x 10^−3^	0.15
	BMI (sex-combined)	1.40 x 10^−38^	9.04 x 10^−38^	3.18 x 10^−4^	1.06 x 10^−3^	0.999
	BMI (female-specific)	3.38 x 10^−7^	2.19 x 10^−6^	3.42 x 10^−4^	1.22 x 10^−3^	0.998
	Comparative body size age 10	2.47 x 10^−8^	1.60 x 10^−7^	3.32 x 10^−4^	1.14 x 10^−3^	0.999
	T2D	4.02 x 10^−10^	2.58 x 10^−9^	0.13	0.86	3.54 x 10^−3^
	T2D (BMI adj.)	1.71 x 10^−9^	1.10 x 10^−8^	0.013	0.087	0.90
2-h GIP	Fasting glucose	9.17 x 10^−4^	5.91 x 10^−3^	0.11	0.69	0.20
	2h glucose	1.08 x 10^−21^	1.47 x 10^−20^	1.56 x 10^−4^	1.13 x 10^−3^	0.999
	HbA_1c_	8.87 x 10^−3^	0.12	0.016	0.21	0.64
	AUC_ins_/AUC_gluc_	0.020	0.27	1.79 x 10^−3^	0.024	0.68
	AUC_ins_	0.020	0.27	4.28 x 10^−4^	5.15 x 10^−3^	0.71
	Incr_30_	5.23 x 10^−3^	0.072	0.047	0.65	0.23
	Ins_30_ (BMI adj.)	0.029	0.39	3.57 x 10^−3^	0.048	0.53
	Ins_30_	2.73 x 10^−3^	0.037	9.37 x 10^−3^	0.13	0.82
	ISI	0.058	0.79	1.81 x 10^−4^	2.32 x 10^−3^	0.15
	BMI (sex-combined)	6.79 x 10^−39^	9.35 x 10^−38^	1.54 x 10^−4^	1.13 x 10^−3^	0.999
	BMI (female-specific)	5.42 x 10^−8^	7.44 x 10^−7^	1.74 x 10^−4^	1.39 x 10^−3^	0.998
	Comparative body size age 10	1.22 x 10^−8^	1.66 x 10^−7^	1.63 x 10^−4^	1.24 x 10^−3^	0.999
	T2D	2.04 x 10^−10^	2.79 x 10^−9^	0.068	0.93	1.19 x 10^−3^
	T2D (BMI adj.)	7.98 x 10^−10^	1.09 x 10^−8^	6.35 x 10^−3^	0.086	0.91

HbA1c = glycated hemoglobin, AUCIns/AUCGluc (mU/mmol) = ratio of the area under the curve (AUC) for AUC insulin/AUC glucose calculated using the trapezium rule; Ins30 = insulin at 30 min; Incr30 = incremental insulin at 30 min, calculated by insulin 30 min - fasting insulin; Ins30 (BMI adj.) = insulin response to glucose during the first 30 min adjusted for BMI, calculated using insulin at 30 min/(glucose at 30 min×BMI); AUCIns (mU∗min/l) = area under the curve (AUC) of insulin levels during oral glucose tolerance test, ISI = Insulin sensitivity index, calculated using 10,000/√ (fasting plasma glucose (mg/dL)×fasting insulin×mean glucose during OGTT (mg/dL)×mean insulin during OGTT), BMI = body mass index, comparative body size at age 10 = recall of an individual’s body size at age 10 as compared to average.

H_0_-H_4_: posterior probabilities of the associations between the 2 traits examined, evaluating 5 different configurations.

H_0_: Neither trait has an association in the region.

H_1_: The first trait has an association in the region but the second does not.

H_2_: The second trait has an association in the region but the first does not.

H3: Both traits have an association in the region but have different causal variants

H4: Both traits have an association in the region and share the same causal variant.

**Figure 4:**
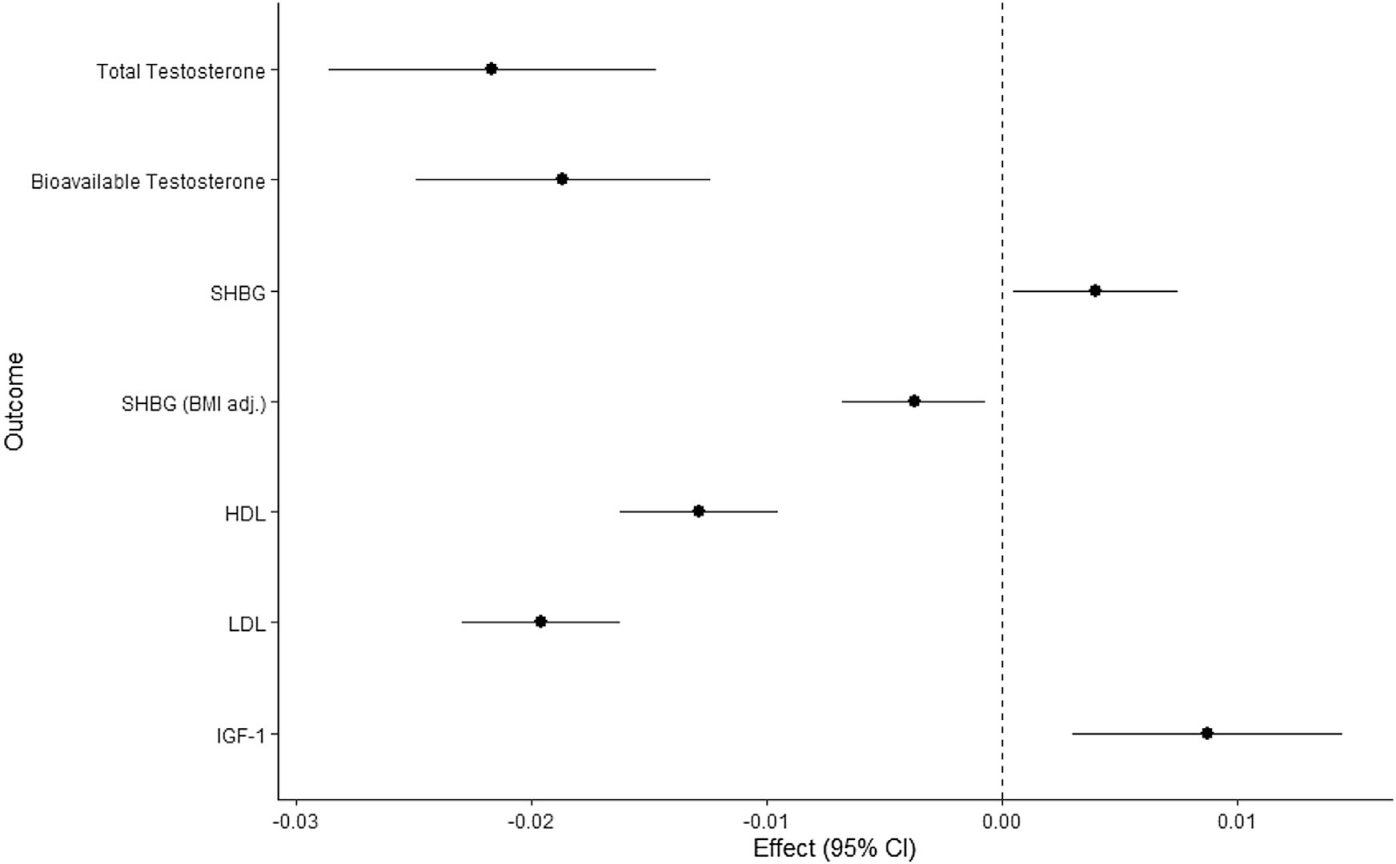
Association between E354Q and sex hormone measures, lipid measures, and IGF-1 Effect represents the change in continuous trait per copy of E354Q (rs1800437, C allele). OR represents the exponential increase in odds per copy of E354Q.BMI = body mass index, SHBG = sex hormone-binding globulin, HDL = high-density lipoprotein, LDL = low-density lipoprotein, IGF-1 = insulin-like growth factor 1.Unit change in each outcome measure is as follows: total testosterone (inverse-normal transformed, nmol/L), bioavailable testosterone (natural log transformed, nmol/L), SHBG (INT, nmol/L), SHBG adjusted for BMI (INT, nmol/L), HDL (SD, mg/dL), LDL (SD, mg/dL), insulin-like growth factor 1 (IGF-1) (inverse-rank normalized, nmol/L).∗These four sex hormone measures were assessed in a female subgroup only.

When examining hormone and lipid traits, there was also consistent MR and colocalization evidence to implicate E354Q in lower total (−0.022, 95% CI:-0.029,-0.015, p = 5.00x10^−10^, H_4_ = 99.8%) and bioavailable testosterone concentrations in women(-0.019, 95% CI:-0.025,-0.012, p = 5.20x10^−9^, H_4_ ≥ 99.5%)(Figure 4, Tables 4 and S4). Full MR and colocalization estimates across all potential mediators examined are presented in Table 2, Table 3, Table 4, S3, and S4. Findings from iterative leave-one-out analysis are presented in Table S5.

**Table 4:** Colocalization analysis results for fasting and 2-h GIP concentrations and sex hormone measures, lipid measures and IGF-1

Exposure	Outcome	H_0_	H_1_	H_2_	H_3_	H_4_
Fasting GIP	SHBG (BMI adj.)	8.75 x10^−41^	5.65 x10^−40^	0.13	0.85	0.018
	Bioavailable testosterone	2.53 x10^−6^	1.63 x10^−5^	8.05 x10^−4^	4.20 x10^−3^	0.995
	Total testosterone	4.53 x10^−7^	2.92 x10^−6^	3.62 x10^−4^	1.34 x10^−3^	0.998
	SHBG	2.53 x10^−27^	1.63 x10^−26^	0.13	8.50 x10^−1^	0.018
	HDL-c	2.91 x10^−29^	1.88 x10^−28^	0.13	8.50 x10^−1^	0.018
	LDL-c	1.02 x10^−48^	6.61 x10^−48^	0.13	8.58 x10^−1^	8.55 x10^−3^
	IGF-1	0.11	0.74	6.48 x10^−4^	4.04 x10^−3^	0.143
2-h GIP	SHBG (BMI adj.)	4.52 x10^−41^	6.18 x10^−40^	0.068	9.31 x10^−1^	1.16 x10^−3^
	Bioavailable testosterone	1.12 x10^−6^	1.54 x10^−5^	3.58 x10^−4^	3.91 x10^−3^	0.996
	Total testosterone	2.22 x10^−7^	3.03 x10^−6^	1.77 x10^−4^	1.43 x10^−3^	0.998
	SHBG	1.31 x10^−27^	1.79 x10^−26^	0.068	9.31 x10^−1^	1.16 x10^−3^
	HDL-c	1.50 x10^−29^	2.05 x10^−28^	0.68	9.31 x10^−1^	1.16 x10^−3^
	LDL-c	5.01 x10^−49^	6.85 x10^−48^	0.065	8.90 x10^−1^	0.045
	IGF-1	0.058	0.79	3.26 x10^−4^	4.31 x10^−3^	0.15

SHBG = sex hormone-binding globulin, HDL = high-density lipoprotein, LDL = low density lipoprotein, IGF-1 = insulin-like growth factor 1.

H_0_-H_4_: posterior probabilities of the associations between the 2 traits examined, evaluating 5 different configurations.

H_0_: Neither trait has an association in the region.

H_1_: The first trait has an association in the region but the second does not.

H_2_: The second trait has an association in the region but the first does not.

H3: Both traits have an association in the region but have different causal variants

H4: Both traits have an association in the region and share the same causal variant.

### Association of traits influenced by E354Q with breast cancer risk

**Figure 5:**
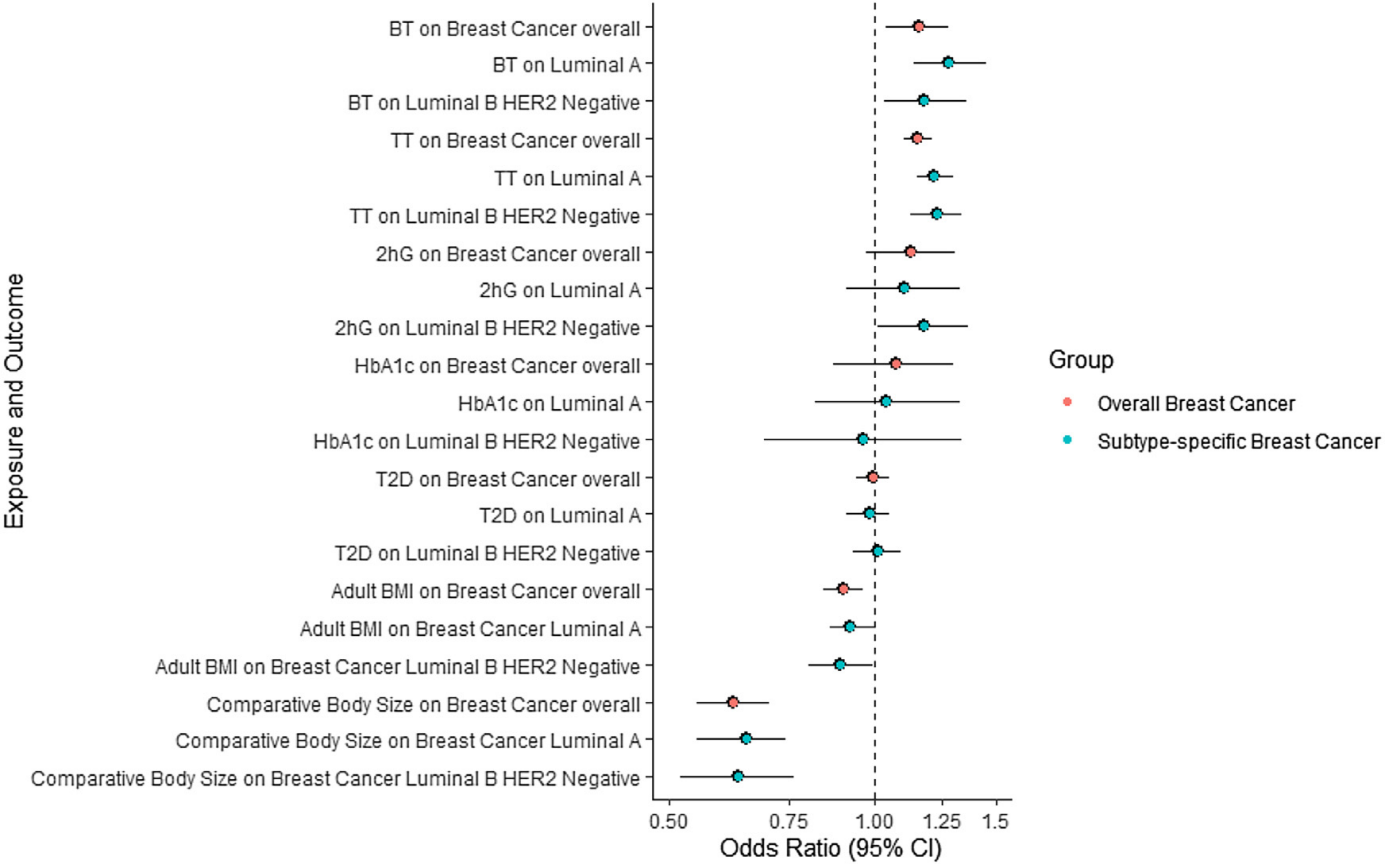
Association between genetically proxied testosterone (bioavailable and total), glucose levels 2 h post OGTT, HbA_1c_, T2DM adjusted for BMI, adult BMI, and comparative body size at age 10 and risk of overall and histotype-specific breast cancer OR represents the exponential increase in odds per copy of E354Q (rs1800437, C allele).BT = Bioavailable testosterone, TT = Total testosterone, BMI = body mass index, 2hrG = glucose concentration measured 2 h after OGTT, HbA_1c_ = glycated hemoglobin.

For putative mediators where there was evidence from MR and colocalization analyses that E354Q influenced that trait, we then evaluated whether there was evidence for an effect of that trait on breast cancer risk. In inverse-variance weighted (IVW) models, genetically proxied bioavailable testosterone was associated with overall (OR:1.16, 95% CI:1.04–1.28, p = 6.53x10^−3^), luminal A-like (OR:1.28, 95% CI:1.14–1.45, p = 5.27x10^−5^), and luminal B HER2 negative-like breast cancer risk (OR:1.18, 95% CI:1.03–1.36, p = 0.02) (Figure 5, Table S6). Likewise, genetically proxied total testosterone was associated with overall (OR:1.15, 95% CI:1.10–1.21, p = 9.39x10^−9^), luminal A-like (OR:1.22, 95% CI:1.15–1.30, p = 5.80x10^−11^), and luminal B HER2 negative-like breast cancer risk (OR:1.23, 95% CI:1.13–1.34, p = 1.02x10^−6^) (Figure 5, Table S6). When employing weighted median and mode models, there was an attenuation of the association of genetically proxied total testosterone with luminal B HER2Neg-like breast cancer risk (Figure 5, Table S6).

We also found evidence that genetically proxied adult BMI was associated with a lower risk of overall (OR:0.90, 95% CI:0.84–0.96, p = 1.08x10^−3^), luminal A-like (OR:0.92, 95% CI:0.86–1.00, p = 0.039), and luminal B HER2 negative-like breast cancer risk (OR:0.89, 95% CI:0.80–0.99, p = 0.040). Genetically proxied smaller comparative body size at age 10 was likewise associated with lower risk of overall (OR:0.62, 95% CI:0.55–0.70, p = 8.25^−14^), luminal A-like (OR:0.65, 95% CI:0.55–0.74, p = 2.19x10^−8^), and luminal B HER2 negative-like breast cancer risk (OR:0.63, 95% CI:0.52–0.76, p = 1.87x10^−6^) (Figure 5, Table S6). However, findings for genetically proxied adult BMI on luminal A breast cancer risk were not consistent in sensitivity analyses (Table S6). There was little evidence for an association of genetically proxied 2-h glucose, HbA_1c_, or genetic liability to type 2 diabetes with breast cancer risk (Figure 5, Table S6). Single-nucleotide polymorphisms (SNPs) excluded in the outlier corrected analysis for the MR-PRESSO are presented in Table S7.

When combining adult BMI and comparative body size at age 10 in a multivariable MR model examining overall and luminal B HER2 negative-like breast cancer risk, the direct effect of adult BMI on breast cancer risk was attenuated for overall and luminal BHER2 negative-like breast cancer risk (overall breast cancer risk OR:1.09, 95% CI:0.99–1.20, p = 0.085) but the direct effect of comparative body size at age 10 was retained for overall and luminal B HER2negative-like breast cancer risk (overall breast cancer risk OR:0.56, 95% CI:0.46–0.67, p = 5.04x10^−10^)(Table S8).

## Discussion

In this MR analysis of up to 235,698 cancer cases and 333,932 controls, each copy of the *GIPR* E354Q missense variant was associated with a higher risk of overall, luminal A-like, and luminal B HER2 negative-like breast cancer risk. These findings were supported in colocalization analysis and were replicated in an independent sample of 8,401 breast cancer cases and 99,321 controls. Although colocalization analyses were performed using fasting GIP concentrations, putative causal effects are unlikely to be driven through fasting GIP concentrations; rather, effects are more likely to reflect the GIPR signaling pathway, of which fasting GIP concentrations are a marker.

E354Q was also associated with higher 2-h glucose concentrations but diminished insulin secretion and lower total and bioavailable testosterone concentrations. These measures confer opposing effects on breast cancer risk, suggesting perturbed glycemic and/or other adverse effects of impaired GIPR signaling through this mechanism offset possible beneficial effects on insulin secretion and circulating testosterone levels. Further work validating these findings and clarifying mechanisms using alternative approaches could help to reconcile these findings. There was little evidence of association of E354Q with the risk of the 5 other cancers examined.

The *GIPR* E354Q variant has previously been implicated in increased Glucose-dependent insulinotropic polypeptide-Glucose-dependent insulinotropic polypeptide receptor (GIP-GIPR) residence time, signaling, internalization and thus likely desensitization and downregulation of the signaling pathway long-term in some tissues.^29^ Consistent with prior studies, each copy of the E354Q variant was associated with various indices of diminished postprandial insulin secretion.^17^^,^^28^^,^^31^ Given the established role of sustained elevated blood insulin levels in the development of breast cancer, the adverse association of E354Q with breast cancer endpoints suggests that this effect is likely mediated via non-insulinemic pathways.^9^ This observation is further reinforced by the specificity of the association of E354Q with breast cancer risk, given important roles of hyperinsulinemia in the 5 other cancers examined in this analysis. Though further experimental work is required to validate and clarify potential mechanisms governing this effect, our findings suggesting an adverse association of E354Q with breast cancer risk provide tentative support for a potential protective effect of enhanced GIPR signaling (i.e. GIPR agonism) on breast cancer risk. Adipokines, including adiponectin and resistin, have previously been linked to breast cancer risk in conventional observational studies and could provide another potential mechanism linking GIPR signaling to breast cancer risk.^32^^,^^33^ However, prior MR analysis suggested that both circulating adiponectin and resistin levels are unlikely to causally influence breast cancer risk and, hence, these measures were not included as potential molecular mediators in this analysis.^34^

Our findings are not consistent with a previous conventional epidemiological analysis which found little evidence of an association of circulating GIP concentrations with breast cancer risk (OR for women at and above vs. below median GIP levels: 1.06, 95% CI:0.63–1.84), though this study was restricted to 109 cancer cases and GIP was measured in non-fasting samples which could result in substantial measurement error.^23^ While preclinical studies suggest that GIP can induce cAMP elevation in medullary thyroid cancer cells and proliferation in colorectal cancer cells, no known *in vitro* or *in vivo* studies have examined the role of GIP signaling in breast cancer to date.^14^^,^^35^

In our analyses, E354Q was associated with lower adult BMI levels which is not consistent with weight loss observed in clinical trials of GIPR agonists (alongside GLP1R agonists).^36^^,^^37^ Interestingly, both GIPR agonists and antagonists have been shown to induce weight loss in preclinical settings.^38^ One possible explanation for this apparent paradox is agonism-induced desensitization of the GIPR, in which persistent stimulation of the GIP receptor by an agonist results in an increasingly diminished response and, consequently, a weight-loss effect.^38^ This theory is supported by preclinical work in adipose cell culture which has demonstrated that GIPR responsiveness is impaired following repeated stimulation, and this repeated stimulation results in downregulation of GIPR at the plasma membrane.^38^^,^^39^

The E354Q variant was also associated with smaller self-reported comparative body size at age 10, but not with measured BMI in children aged 2–10. In univariable MR models, both adult BMI and smaller self-reported comparative body size at age 10 were associated with lower breast cancer risk, though only childhood smaller self-reported comparative body size showed evidence of a direct effect on breast cancer in multivariable MR models, consistent with prior MR analysis.^40^ Consistent with a recent meta-analysis of 37 prospective studies, our findings suggest a protective association of higher early life BMI with breast cancer risk.^41^ It is therefore plausible that part of a potential adverse effect of E354Q on breast cancer risk is mediated via lower early life adiposity, though discrepancies in findings between smaller self-reported comparative body size and measured BMI in childhood require further exploration in future studies.

There was little evidence of association of E354Q with the risk of the 5 other cancers examined, which could reflect the relatively smaller sample sizes and, consequently, lower power for these other cancer sites. Alternatively, the specificity of the association of E354Q with breast cancer risk could reflect a potentially unique role of GIPR signaling in breast carcinogenesis. Our findings suggest that a potential adverse effect of impaired GIPR signaling on breast cancer risk is unlikely to be mediated via insulinemic and/or hormonal pathways. Along with further evaluation of the potential mediating role of lower childhood adiposity in this relationship, evaluation of the effect of pharmacological GIPR perturbation in breast cancer cell lines and/or animal models could provide further insight into potential mechanisms governing this effect.

Strengths of this analysis include the use of an MR approach, which should be less susceptible to issues of confounding and reverse causation than conventional epidemiological analyses; the use of a summary-data MR approach which permitted use to leverage data from several large genome-wide association study (GWAS) consortia, increasing statistical power and precision of causal estimates; and the comprehensive assessment of the effect of GIPR signaling across a large panel of glycemic, hormonal, and lipidomic mediators which enabled us to evaluate potential biological mechanisms through which impaired GIPR signaling may confer an increased risk of breast cancer.

There is considerable interest in the pharmacological modification of GIPR signaling as treatment for type 2 diabetes and obesity. Our findings, using an established missense variant in *GIPR* to proxy impaired GIPR signaling, suggest potential adverse effects of downregulated GIPR signaling on breast cancer risk and, thus, possible protective effects of pharmacological GIPR agonism. Given the sparsity of preclinical and epidemiological literature examining the role of GIPR signaling in breast cancer development, further work is warranted to validate and clarify potential mechanisms underpinning this putative effect. In particular, further evaluation of possible non-insulinemic pathways influenced by GIPR signaling could help to reconcile the specificity of the E354 association with breast cancer risk given the important role of metabolic dysfunction across the 5 other cancers examined in this analysis. Though clinical trial data support the efficacy of dual GIPR/GLP1R agonism for glycemic control in type 2 diabetes, it is unclear whether pharmacological GIPR agonism alone would confer similar favorable effects on glucose metabolism.^36^^,^^38^ Evaluation of the role of genetically proxied GLP1R signaling, alone and in combination with genetically proxied GIPR signaling, could provide additional insight into the viability of dual pharmacological GLP1R/GIPR agonism for breast cancer prevention.

In conclusion, our drug-target MR analyses across 6 cancers suggest adverse effects of the *GIPR* E354Q missense variant on breast cancer risk. In mechanistic analyses, this variant was associated with higher levels of 2-h glucose but diminished insulin secretion and lower total and bioavailable testosterone concentrations. Triangulation of these findings in other settings will inform on the efficacy of pharmacologically modifying GIPR signaling as a potential chemoprevention strategy for breast cancer.^42^

### Limitations of the study

There are several limitations to these analyses. First, drug-target MR analyses are restricted to examining the “on-target” effects of pharmacological interventions. Second, the effect estimates presented assume linear and time-fixed effects of GIPR signaling and the absence of gene-environment and gene-gene interactions. Third, MR analyses consider the small, lifelong effects exerted by a genetic variant, which may not necessarily translate to the clinical effect observed through pharmacological intervention in adult life. Fourth, statistical power was likely limited for some less common cancer sites (e.g. pancreatic and renal cancer) and histological subtypes (e.g. small cell lung cancer). Statistical power can also often be limited in colocalization analyses which can reduce the likelihood of shared causal variants across traits being detected. Fifth, we were unable to examine the effect of four measures of insulin secretion (AUC_ins_/AUC_gluc_, AUC_ins_, Ins_30_, and Ins_30_ [BMI adj.]), influenced by E354Q, on breast cancer risk due to the lack of genome-wide significant variants available to serve as instruments for these measures. Furthermore, we were unable to directly test the effects of estrogen and progesterone on breast cancer risk due to a lack of robust instruments for these traits. Sixth, effect estimates were generated from data on participants without type 2 diabetes and therefore findings may not generalize to those with this condition. In addition, our findings did not recapitulate the known weight-loss effect of tirzepatide, which we believe is driven by receptor desensitization, though this could not be verified by the data available to us. Furthermore, while the restriction of participants to those of European ancestry, the use of a functional variant in *GIPR* to instrument GIPR signaling, and the use of colocalization should help to minimize exchangeability and exclusion restriction violations, these assumptions are unverifiable. In addition, our use of a single genetic variant to instrument GIPR signaling prevented us from employing various pleiotropy-robust methods to evaluate and/or mitigate the presence of horizontal pleiotropy. We selected 50% as a posterior probability threshold for colocalization of traits given the low statistical power of this analysis and the limited power for some anatomical site/subtype-specific cancer analyses. We cannot rule out the possibility that the use of a more liberal threshold to account for the limited power of these analyses may have meant that some traits reported as "colocalized" may represent alternate SNP association patterns in *GIPR*, such as distinct causal variants influencing traits or only one of two traits having a causal variant in this locus.

## STAR★Methods

### Key resources table

**Table:** 

REAGENT or RESOURCE	SOURCE	IDENTIFIER
**Software and algorithms**		

PLINK	Purcell et al.^43^	http://pngu.mgh.harvard.edu/purcell/plink/
LocusZoom	Boughton et al.^44^	http://locuszoom.org
Coloc R package	Giambartolomei et al.^45^	coloc package - RDocumentation
TwoSampleMR package	Hemani et al.^46^	Two Sample MR Functions and Interface to MR Base Database · TwoSampleMR (mrcieu.github.io)
MR-PRESSO	Verbanck et al.^47^	GitHub - rondolab/MR-PRESSO: Performs the Mendelian Randomization Pleiotropy RESidual Sum and Outlier (MR-PRESSO) method.
Contamination Mixture model	Burgess et al.^48^	mr_conmix: Contamination mixture method in MendelianRandomization: Mendelian Randomization Package (rdrr.io)

**Other**		

Summary genetic association data: breast cancer	Zhang et al.^49^	https://bcac.ccge.medschl.cam.ac.uk/bcacdata/
Summary genetic association data: breast cancer in BRCA1/2 mutation carriers	Phelan et al.^50^ Milne et al.^51^	https://cimba.ccge.medschl.cam.ac.uk/projects/
Summary genetic association data: endometrial cancer	O’Mara et al.^52^	https://www.ebi.ac.uk/gwas
Summary genetic association data: lung cancer	Wang et al.^53^	https://www.ebi.ac.uk/gwas/
Summary genetic association data: pancreatic cancer	Klein et al.^54^	Obtained via dbGaP release phs000206.v5.p3
Summary genetic association data: colorectal cancer	Huyghe et al.^55^	Accessed by contacting GECCO (kafdem@fredhutch.org)
Summary genetic association data: Finngen consortium	Kurki et al.^56^	https://www.finngen.fi/en/access_results
Summary genetic association data: MAGIC consortium	Prokopenko et al.^57^	https://magicinvestigators.org/downloads/
Summary genetic association data: GIANT consortium	Locke et al.^58^	https://portals.broadinstitute.org/collaboration/giant/index.php/GIANT_consortium
Summary genetic association data: DIAGRAM consortium	Mahajan et al.^59^	https://diagram-consortium.org/downloads.html
Summary genetic association data: UK Biobank-derived traits	IEU GWAS catalog^60^	Accessed via the IEU Open GWAS project (https://gwas.mrcieu.ac.uk/).

### Resource availability

#### Lead contact

Further information and requests for resources should be directed to and will be fulfilled by the lead contact, James Yarmolinsky (james.yarmolinsky@bristol.ac.uk)

#### Materials availability

This study did not generate any new unique reagents.

### Experimental model and subject details

#### Study population

Summary genetic association data on overall and histological subtype-specific cancer susceptibility were obtained from genome-wide association study (GWAS) meta-analyses of 6 adult cancers in up to 235,698 cases and 333,932 controls of European ancestry. Cancer sites were selected based on previous genetic epidemiological evidence linking fasting insulin to cancer susceptibility and included the following anatomical sites: breast (133,384 cases, 113,789 controls), colorectum (58,221 cases, 67,694 controls), endometrium (12,906 cases, 108,979 controls), lung (11,348 cases, 15,861 controls), kidney (10,784 cases, 20,406 controls), and pancreas (9,055 cases, 7,203 controls).^3^^,^^4^^,^^5^^,^^7^^,^^8^^,^^9^^,^^49^^,^^52^^,^^53^^,^^54^^,^^55^^,^^61^ Further information on numbers of cases and controls across histological subtype-stratified analyses is presented in Table S2.

For replication analyses, summary genetic association data were obtained on 8,401 breast cancer cases and 99,321 controls of European ancestry from the Finngen consortium.^56^ We also performed exploratory analyses examining the association of impaired GIPR signalling with breast cancer risk in *BRCA1/2* mutation carriers, by obtaining GWAS summary data on 19,306 *BRCA1* mutation carriers (of whom 7,502 did not develop breast or ovarian cancer; 2,009 developed ovarian cancer only; 8,601 developed breast cancer only, and 924 developed breast and ovarian cancer) and 12,412 *BRCA2* mutation carriers (of whom 5,354 did not develop breast or ovarian cancer; 692 developed ovarian cancer only; 6,104 developed breast cancer only; and 262 developed breast and ovarian cancer) of European ancestry from the Breast Cancer Association Consortium (BCAC) and Consortium of Investigations of Modifiers of BRCA1/2 (CIMBA).^50^^,^^51^

For analyses investigating the effect of impaired GIPR signalling on putative mediators of the GIPR-breast cancer relationship, we obtained summary genetic association data from previous GWAS of child and adult BMI or smaller self-reported comparative body size, type 2 diabetes, 3 endogenous sex hormones, 4 glycaemic traits measured in the non-postprandial state, 11 glycaemic traits measured following an oral glucose tolerance test, 2 lipid traits, and insulin-like growth factor 1.^57^^,^^58^^,^^59^^,^^60^^,^^62^^,^^63^^,^^64^^,^^65^^,^^66^^,^^67^^,^^68^ These traits were selected based on previous observational and genetic epidemiological evidence supporting their potential role in breast cancer risk.^64^^,^^69^^,^^70^^,^^71^^,^^72^^,^^73^ Data on endogenous sex hormone were restricted to analyses performed in women. All 14 glycaemic traits were measured in non-diabetic individuals. Following suggestions made in peer-review, we also examined the association of impaired GIPR signalling with circulating glucagon.^74^ Additional information on the specific traits included, their measurement, along with participant characteristics and covariates included in adjustment strategies across each GWAS are presented in Table S9. Further information on imputation, statistical analyses and quality control measures for these studies can be found in the original publications.

### Method details

#### Instrument construction

We used a missense variant in *GIPR*, rs1800437 (E354Q, C allele), to proxy impaired GIPR signalling. This variant has been implicated in increased GIP residence time at GIPR, increased internalisation and signalling, and thus desensitisation and impairment of the signalling pathway long-term.^29^ This variant was also associated (*P*<5.0x10^-8^) with lower fasting and 2-hour GIP concentrations in a GWAS meta-analysis of 7,828 individuals of European ancestry across the Malmö Diet and Cancer (MDC) and Prevalence, Prediction and Prevention of diabetes (PPP)-Botnia studies. Participants in both studies were not taking anti-diabetic medications.^28^ Summary genetic association data on fasting and 2-hour GIP concentrations were obtained from the MDC subcohort because of denser variant coverage as compared to the PPP-Botnia study.

To generate genetic instruments to proxy potential mediators of the GIPR signalling-cancer relationship, genome-wide significant (*P*<5.0x10^-8^) and independent (r^2^<0.001) SNPs were selected using the 1000 Genomes Phase 3 European reference panel.^43^

### Quantification and statistical analysis

Analyses of the effect of traits influenced by E354Q on cancer risk (i.e. putative mediators of the effect of E354Q on cancer risk) were performed using inverse-variance weighted (IVW) random-effects models.^75^

Mendelian randomization (MR) analysis assumes that a genetic instrument (i) is associated with a modifiable exposure or drug target (“relevance”), (ii) does not share a common cause with an outcome (“exchangeability”), and (iii) has no direct effect on the outcome (“exclusion restriction”).^76^^,^^77^ Under the assumption of monotonicity (i.e. the direction of effect of the instrument on the exposure is consistent across all individuals), MR can provide valid point estimates for those participants whose exposure is influenced by the instrument (i.e. a local average treatment effect^78^).

We assessed the “relevance” assumption by generating estimates of the proportion of variance in each trait explained by the instrument (r^2^) and F-statistics. An F-statistic >10 is conventionally used to indicate that instruments are unlikely to suffer from weak instrument bias.^30^

Colocalisation was performed as a sensitivity analysis for primary analyses where there was nominal evidence of an association (*P*<0.05), to assess whether two traits examined shared a causal variant at a genetic locus (e.g. as opposed to both traits having distinct causal variants that are in linkage disequilibrium).^45^ Colocalisation analyses were performed using the coloc R package by generating ±250 kb windows from the sentinel SNP used to proxy the instrument.^45^ We used H_4_>50.0% as evidence to support colocalisation of traits.

When testing the effect of putative GIPR signalling-cancer mediators on cancer risk, we evaluated the “exclusion restriction” assumption through performing various sensitivity analyses, including MR-Egger, weighted median, weighted mode, MR-PRESSO and contamination mixture models.^76^^,^^77^^,^^78^^,^^46^^,^^47^^,^^48^ We also performed iterative “leave-one-out” analysis to examine the robustness of findings to individual influential SNPs in IVW models.

To account for multiple testing across E354Q-cancer analyses, a Bonferroni correction was used to establish a *P*-value threshold of <0.0029 (false positive rate=0.05/17 statistical tests, representing 17 cancer endpoints), which we used as a heuristic to define “strong evidence,” with findings between *P*≥0.0029 and *P*<0.05 defined as “weak evidence.”

### Additional resources

• PLINK: http://pngu.mgh.harvard.edu/purcell/plink/.^43^
• LocusZoom: LocusZoom - Create Plots of Genetic Data.^44^

## Data Availability

• Genetic association data were obtained from different sources/consortia. Full source and consortia information is in the key resources table. • All data reported in this paper will be shared by the lead contact upon request. • This paper does not report original code. • Any additional information required to reanalyze the data reported in this paper is available from the lead contact upon request.

## Supplemental information

Table S1. Characteristics of genetic variants used to proxy GIPR signaling and putative mediators, related to Figures 1–5

Table S2. Association between E354Q and overall and histotype-specific breast, endometrial, colorectal, lung, renal, and pancreatic cancer risk, related to Figure 1 OR represents the exponential increase in odds per copy of E354Q (rs1800437, C allele). ∗Effective sample size not available.

Table S3. Association between E354Q and glycemic traits, adiposity measures, and type 2 diabetes (adjusted and unadjusted for BMI), related to Figures 2 and 3 Effect estimate represents the change in continuous trait per copy of E354Q (rs1800437, C allele). OR represents the exponential increase in odds per copy of E354Q. HbA1c = glycated hemoglobin, CIR = Corrected Insulin Response, calculated using 100× insulin at 30 min)/(glucose at 30 min×(glucose at 30 min–3.89); AUC_Ins_/AUC_Gluc_ (mU/mmol) = ratio of the area under the curve (AUC) for AUC insulin/AUC glucose calculated using the trapezium rule; ISI = Insulin sensitivity index, calculated using 10,000/√ (fasting plasma glucose (mg/dL)×fasting insulin×mean glucose during OGTT (mg/dL)×mean insulin during OGTT); CIR_ISI = CIR adjusted for insulin sensitivity index; DI = disposition index, calculated using CIR×ISI; Ins_30_ = insulin at 30 min; Incr_30_ = incremental insulin at 30 min, calculated by insulin 30 min – fasting insulin; Ins_30_ (BMI adj.) = insulin response to glucose during the first 30 min adjusted for BMI, calculated using insulin at 30 min/(glucose at 30 min×BMI); AUCIns (mU∗min/l) = area under the curve (AUC) of insulin levels during OGTT, HOMA-IR = Homeostatic model assessment of insulin resistance, HOMA-B = Homeostatic model assessment of beta-cell function, T2D = type 2 diabetes, BMI = body mass index (adult), childhood BMI = BMI in children aged between 2 and 10 years old, comparative body size at age 10 = Recall of an individual’s body size at age 10 as compared to average. Glucagon levels were from random plasma sample.

Table S4. Association between E354Q and sex hormone measures, lipid measures, and IGF-1, related to Figure 4 BMI = body mass index, SHBG = sex hormone-binding globulin, HDL = high-density lipoprotein, LDL = low-density lipoprotein, IGF-1 = Insulin-like growth factor 1. ∗These four sex hormone measures were assessed in a female subgroup only.

Table S5. Leave-one-out analyses, related to Figure 5

Table S6. Association between genetically proxied testosterone (bioavailable and total), glucose levels 2 h post OGTT, HbA1c, T2DM adjusted for BMI, adult BMI, and comparative body size at age 10 and risk of overall and histotype-specific breast cancer, related to Figure 5 BMI = body mass index, 2hrG = glucose concentration measured 2 h after OGTT, HbA_1c_ = glycated hemoglobin. SNPs excluded in each MR-PRESSO outlier-corrected analysis are presented in Table S7.

Table S7. Outlier SNPs removed when conducting outlier-correct analyses for MR-PRESSO analysis of the association of traits influenced by E354Q with breast cancer risk, related to Figure 5

Table S8. MVMR results for adult BMI and comparative body size at age 10 on breast cancer risk (by histotype), related to Figure 5

Table S9. Participant characteristics and covariates included in adjustment strategies across each GWAS for each trait examined, related to Figures 1–5 ∗There were differing study level adjustment stategies between studies used for most traits. Only those that were present in all studies have been listed.

## References

[1] Onitilo A.A., Engel J.M., Glurich I., Stankowski R.V., Williams G.M., Doi S.A. (2012). Diabetes and cancer II: role of diabetes medications and influence of shared risk factors. Cancer Causes Control.

[2] Shikata K., Ninomiya T., Kiyohara Y. (2013). Diabetes mellitus and cancer risk: review of the epidemiological evidence. Cancer Sci..

[3] Murphy N., Song M., Papadimitriou N., Carreras-Torres R., Langenberg C., Martin R.M., Tsilidis K.K., Barroso I., Chen J., Frayling T. (2022). Associations between glycemic traits and colorectal cancer: a mendelian randomization analysis. Jnci. J. Natl. Cancer Inst..

[4] Johansson M., Carreras-Torres R., Scelo G., Purdue M.P., Mariosa D., Muller D.C., Timpson N.J., Haycock P.C., Brown K.M., Wang Z. (2019). The influence of obesity-related factors in the etiology of renal cell carcinoma—a mendelian randomization study. PLoS Med..

[5] Carreras-Torres R., Johansson M., Gaborieau V., Haycock P.C., Wade K.H., Relton C.L., Martin R.M., Davey Smith G., Brennan P. (2017). The role of obesity, type 2 diabetes, and metabolic factors in pancreatic cancer: a mendelian randomization study. J. Natl. Cancer Inst..

[6] Gunter M.J., Hoover D.R., Yu H., Wassertheil-Smoller S., Rohan T.E., Manson J.E., Li J., Ho G.Y.F., Xue X., Anderson G.L. (2009). Insulin, insulin-like growth factor-I, and risk of breast cancer in postmenopausal women. J. Natl. Cancer Inst..

[7] Nead K.T., Sharp S.J., Thompson D.J., Painter J.N., Savage D.B., Semple R.K., Barker A., Attia J., Perry J.R.B., Australian National Endometrial Cancer Study Group ANECS (2015). Evidence of a causal association between insulinemia and endometrial cancer: a mendelian randomization analysis. J. Natl. Cancer Inst..

[8] Carreras-Torres R., Johansson M., Haycock P.C., Wade K.H., Relton C.L., Martin R.M., Davey Smith G., Albanes D., Aldrich M.C., Andrew A. (2017). Obesity, metabolic factors and risk of different histological types of lung cancer: a Mendelian randomization study. PLoS One.

[9] Shu X., Wu L., Khankari N.K., Shu X.-O., Wang T.J., Michailidou K., Bolla M.K., Wang Q., Dennis J., Milne R.L. (2019). Associations of obesity and circulating insulin and glucose with breast cancer risk: a Mendelian randomization analysis. Int. J. Epidemiol..

[10] Gallagher E.J., LeRoith D. (2020). Hyperinsulinaemia in cancer. Nat. Rev. Cancer.

[11] McIntosh C.H.S., Widenmaier S., Kim S. (2009). Vitamins & Hormones Insulin and IGFs.

[12] Frías J.P., Davies M.J., Rosenstock J., Pérez Manghi F.C., Fernández Landó L., Bergman B.K., Liu B., Cui X., Brown K., SURPASS-2 Investigators (2021). Tirzepatide versus semaglutide once weekly in patients with type 2 diabetes. N. Engl. J. Med..

[13] Mullard A. (2022). Lilly’s tirzepatide secures first approval in diabetes, paving path for dual-acting hormone mimetics. Nat. Rev. Drug Discov..

[14] Prabakaran D., Wang B., Feuerstein J.D., Sinclair J.A., Bijpuria P., Jepeal L.I., Wolfe M.M. (2010). Glucose-dependent insulinotropic polypeptide stimulates the proliferation of colorectal cancer cells. Regul. Pept..

[15] Torekov S.S., Harsløf T., Rejnmark L., Eiken P., Jensen J.B., Herman A.P., Hansen T., Pedersen O., Holst J.J., Langdahl B.L. (2014). A functional amino acid substitution in the glucose-dependent insulinotropic polypeptide receptor (GIPR) gene is associated with lower bone mineral density and increased fracture risk. J. Clin. Endocrinol. Metab..

[16] Jujić A., Atabaki-Pasdar N., Nilsson P.M., Almgren P., Hakaste L., Tuomi T., Berglund L.M., Franks P.W., Holst J.J., Prasad R.B. (2020). Glucose-dependent insulinotropic peptide and risk of cardiovascular events and mortality: a prospective study. Diabetologia.

[17] Bowker N., Hansford R., Burgess S., Foley C.N., Auyeung V.P.W., Erzurumluoglu A.M., Stewart I.D., Wheeler E., Pietzner M., Gribble F. (2021). Genetically predicted glucose-dependent insulinotropic polypeptide (GIP) levels and cardiovascular disease risk are driven by distinct causal variants in the GIPR region. Diabetes.

[18] Jujić A., Nilsson P.M., Atabaki-Pasdar N., Dieden A., Tuomi T., Franks P.W., Holst J.J., Torekov S.S., Ravassa S., Díez J. (2021). Glucose-dependent insulinotropic peptide in the high-normal range is associated with increased carotid intima-media thickness. Diabetes Care.

[19] Hyltén-Cavallius L., Iepsen E.W., Wewer Albrechtsen N.J., Svendstrup M., Lubberding A.F., Hartmann B., Jespersen T., Linneberg A., Christiansen M., Vestergaard H. (2017). Patients with long-QT syndrome caused by impaired hERG-encoded Kv11.1 potassium channel have exaggerated endocrine pancreatic and incretin function associated with reactive hypoglycemia. Circulation.

[20] Møller C.L., Vistisen D., Færch K., Johansen N.B., Witte D.R., Jonsson A., Pedersen O., Hansen T., Lauritzen T., Jørgensen M.E. (2016). Glucose-dependent insulinotropic polypeptide is associated with lower low-density lipoprotein but unhealthy fat distribution, independent of insulin: the ADDITION-PRO study. J. Clin. Endocrinol. Metab..

[21] Wang Q., Tu H., Zhu M., Liang D., Ye Y., Chang D.W., Long Y., Wu X. (2019). Circulating obesity-driven biomarkers are associated with risk of clear cell renal cell carcinoma: a two-stage, case-control study. Carcinogenesis.

[22] Škrha J., Bušek P., Uhrová J., Hrabal P., Kmochová K., Laclav M., Bunganič B., Frič P. (2017). Lower plasma levels of glucose-dependent insulinotropic peptide (GIP) and pancreatic polypeptide (PP) in patients with ductal adenocarcinoma of the pancreas and their relation to the presence of impaired glucoregulation and weight loss. Pancreatology.

[23] Shen J., Hernandez D., Ye Y., Wu X., Chow W.-H., Zhao H. (2019). Metabolic hormones and breast cancer risk among Mexican American women in the mano a mano Cohort study. Sci. Rep..

[24] Holmes M.V., Richardson T.G., Ference B.A., Davies N.M., Davey Smith G. (2021). Integrating genomics with biomarkers and therapeutic targets to invigorate cardiovascular drug development. Nat. Rev. Cardiol..

[25] Smith G.D., Ebrahim S. (2003). ‘Mendelian randomization’: can genetic epidemiology contribute to understanding environmental determinants of disease?. Int. J. Epidemiol..

[26] Yarmolinsky J., Wade K.H., Richmond R.C., Langdon R.J., Bull C.J., Tilling K.M., Relton C.L., Lewis S.J., Davey Smith G., Martin R.M. (2018). Causal inference in cancer epidemiology: what is the role of mendelian randomization?. Cancer Epidemiol. Biomarkers Prev..

[27] Walker V.M., Davey Smith G., Davies N.M., Martin R.M. (2017). Mendelian randomization: a novel approach for the prediction of adverse drug events and drug repurposing opportunities. Int. J. Epidemiol..

[28] Almgren P., Lindqvist A., Krus U., Hakaste L., Ottosson-Laakso E., Asplund O., Sonestedt E., Prasad R.B., Laurila E., Orho-Melander M. (2017). Genetic determinants of circulating GIP and GLP-1 concentrations. JCI Insight.

[29] Gabe M.B.N., van der Velden W.J.C., Gadgaard S., Smit F.X., Hartmann B., Bräuner-Osborne H., Rosenkilde M.M. (2020). Enhanced agonist residence time, internalization rate and signalling of the GIP receptor variant [E354Q] facilitate receptor desensitization and long-term impairment of the GIP system. Basic Clin. Pharmacol. Toxicol..

[30] Staiger, D., and Stock’, J.H. Instrumental variables regression with weak instruments. 30

[31] Saxena R., Hivert M.-F., Langenberg C., Tanaka T., Pankow J.S., Vollenweider P., Lyssenko V., Bouatia-Naji N., Dupuis J., Jackson A.U. (2010). Genetic variation in GIPR influences the glucose and insulin responses to an oral glucose challenge. Nat. Genet..

[32] Vona-Davis L., Rose D.P. (2007). Adipokines as endocrine, paracrine, and autocrine factors in breast cancer risk and progression. Endocr. Relat. Cancer.

[33] Macis D., Guerrieri-Gonzaga A., Gandini S. (2014). Circulating adiponectin and breast cancer risk: a systematic review and meta-analysis. Int. J. Epidemiol..

[34] Robinson T., Martin R.M., Yarmolinsky J. (2020). Mendelian randomisation analysis of circulating adipokines and C-reactive protein on breast cancer risk. Int. J. Cancer.

[35] Regazzo D., Bertazza L., Galletta E., Barollo S., Mondin A., Zovato S., Iacobone M., Zilio E., Scaroni C., Radu C.M. (2022). The GIP/GIPR axis in medullary thyroid cancer: clinical and molecular findings. Endocr. Relat. Cancer.

[36] Frias J.P., Nauck M.A., Van J., Kutner M.E., Cui X., Benson C., Urva S., Gimeno R.E., Milicevic Z., Robins D., Haupt A. (2018). Efficacy and safety of LY3298176, a novel dual GIP and GLP-1 receptor agonist, in patients with type 2 diabetes: a randomised, placebo-controlled and active comparator-controlled phase 2 trial. Lancet.

[37] Jastreboff A.M., Aronne L.J., Ahmad N.N., Wharton S., Connery L., Alves B., Kiyosue A., Zhang S., Liu B., Bunck M.C. (2022). Tirzepatide once weekly for the treatment of obesity. N. Engl. J. Med..

[38] Killion E.A., Lu S.-C., Fort M., Yamada Y., Véniant M.M., Lloyd D.J. (2020). Glucose-dependent insulinotropic polypeptide receptor therapies for the treatment of obesity, do agonists = antagonists?. Endocr. Rev..

[39] Mohammad S., Patel R.T., Bruno J., Panhwar M.S., Wen J., McGraw T.E. (2014). A naturally occurring GIP receptor variant undergoes enhanced agonist-induced desensitization, which impairs GIP control of adipose insulin sensitivity. Mol. Cell Biol..

[40] Richardson T.G., Sanderson E., Elsworth B., Tilling K., Davey Smith G. (2020). Use of genetic variation to separate the effects of early and later life adiposity on disease risk: mendelian randomisation study. BMJ.

[41] Byun D., Hong S., Ryu S., Nam Y., Jang H., Cho Y., Keum N., Oh H. (2022). Early-life body mass index and risks of breast, endometrial, and ovarian cancers: a dose–response meta-analysis of prospective studies. Br. J. Cancer.

[42] Lawlor D.A., Tilling K., Davey Smith G. (2016). Triangulation in aetiological epidemiology. Int. J. Epidemiol..

[43] Purcell S., Neale B., Todd-Brown K., Thomas L., Ferreira M.A.R., Bender D., Maller J., Sklar P., de Bakker P.I.W., Daly M.J., Sham P.C. (2007). PLINK: a tool set for whole-genome association and population-based linkage analyses. Am. J. Hum. Genet..

[44] Boughton A.P., Welch R.P., Flickinger M., VandeHaar P., Taliun D., Abecasis G.R., Boehnke M. (2021). LocusZoom.js: interactive and embeddable visualization of genetic association study results. Bioinformatics.

[45] Giambartolomei C., Vukcevic D., Schadt E.E., Franke L., Hingorani A.D., Wallace C., Plagnol V. (2014). Bayesian test for colocalisation between pairs of genetic association studies using summary statistics. PLoS Genet..

[46] Hemani G., Zheng J., Elsworth B., Wade K.H., Haberland V., Baird D., Laurin C., Burgess S., Bowden J., Langdon R. (2018). The MR-Base platform supports systematic causal inference across the human phenome. Elife.

[47] Verbanck M., Chen C.-Y., Neale B., Do R. (2018). Detection of widespread horizontal pleiotropy in causal relationships inferred from Mendelian randomization between complex traits and diseases. Nat. Genet..

[48] Burgess S., Foley C.N., Allara E., Staley J.R., Howson J.M.M. (2020). A robust and efficient method for Mendelian randomization with hundreds of genetic variants. Nat. Commun..

[49] Zhang H., Ahearn T.U., Lecarpentier J., Barnes D., Beesley J., Qi G., Jiang X., O’Mara T.A., Zhao N., Bolla M.K. (2020). Genome-wide association study identifies 32 novel breast cancer susceptibility loci from overall and subtype-specific analyses. Nat. Genet..

[50] Phelan C.M., Kuchenbaecker K.B., Tyrer J.P., Kar S.P., Lawrenson K., Winham S.J., Dennis J., Pirie A., Riggan M.J., Chornokur G. (2017). Identification of twelve new susceptibility loci for different histotypes of epithelial ovarian cancer. Nat. Genet..

[51] Milne R.L., Kuchenbaecker K.B., Michailidou K., Beesley J., Kar S., Lindström S., Hui S., Lemaçon A., Soucy P., Dennis J. (2017). Identification of ten variants associated with risk of estrogen-receptor-negative breast cancer. Nat. Genet..

[52] O’Mara T.A., Glubb D.M., Amant F., Annibali D., Ashton K., Attia J., Auer P.L., Beckmann M.W., Black A., Bolla M.K. (2018). Identification of nine new susceptibility loci for endometrial cancer. Nat. Commun..

[53] Wang Y., McKay J.D., Rafnar T., Wang Z., Timofeeva M.N., Broderick P., Zong X., Laplana M., Wei Y., Han Y. (2014). Rare variants of large effect in BRCA2 and CHEK2 affect risk of lung cancer. Nat. Genet..

[54] Klein A.P., Wolpin B.M., Risch H.A., Stolzenberg-Solomon R.Z., Mocci E., Zhang M., Canzian F., Childs E.J., Hoskins J.W., Jermusyk A. (2018). Genome-wide meta-analysis identifies five new susceptibility loci for pancreatic cancer. Nat. Commun..

[55] Huyghe J.R., Bien S.A., Harrison T.A., Kang H.M., Chen S., Schmit S.L., Conti D.V., Qu C., Jeon J., Edlund C.K. (2019). Discovery of common and rare genetic risk variants for colorectal cancer. Nat. Genet..

[56] Kurki M., Karjalainen J., Palta P., Sipilä T., Kristiansson K., Donner K., Reeve M., Laivuori H., Aavikko M., Kaunisto M. (2022). FinnGen: unique genetic insights from combining isolated population and national health register data. medRxiv.

[57] Prokopenko I., Poon W., Mägi R., Prasad B R., Salehi S.A., Almgren P., Osmark P., Bouatia-Naji N., Wierup N., Fall T. (2014). A central role for GRB10 in regulation of islet function in man. PLoS Genet..

[58] Locke A.E., Kahali B., Berndt S.I., Justice A.E., Pers T.H., Day F.R., Powell C., Vedantam S., Buchkovich M.L., Yang J. (2015). Genetic studies of body mass index yield new insights for obesity biology. Nature.

[59] Mahajan A., Wessel J., Willems S.M., Zhao W., Robertson N.R., Chu A.Y., Gan W., Kitajima H., Taliun D., Rayner N.W. (2018). Refining the accuracy of validated target identification through coding variant fine-mapping in type 2 diabetes. Nat. Genet..

[60] IEU GWAS QC Report. https://gwas.mrcieu.ac.uk/files/ukb-b-19953/ukb-b-19953_report.html.

[61] Scelo G., Purdue M.P., Brown K.M., Johansson M., Wang Z., Eckel-Passow J.E., Ye Y., Hofmann J.N., Choi J., Foll M. (2017). Genome-wide association study identifies multiple risk loci for renal cell carcinoma. Nat. Commun..

[62] Vogelezang S., Bradfield J.P., Ahluwalia T.S., Curtin J.A., Lakka T.A., Grarup N., Scholz M., van der Most P.J., Monnereau C., Stergiakouli E. (2020). Novel loci for childhood body mass index and shared heritability with adult cardiometabolic traits. PLoS Genet..

[63] IEU GWAS QC Report. https://gwas.mrcieu.ac.uk/files/ukb-b-4650/ukb-b-4650_report.html.

[64] Ruth K.S., Day F.R., Tyrrell J., Thompson D.J., Wood A.R., Mahajan A., Beaumont R.N., Wittemans L., Martin S., Busch A.S. (2020). Using human genetics to understand the disease impacts of testosterone in men and women. Nat. Med..

[65] Chen J., Spracklen C.N., Marenne G., Varshney A., Corbin L.J., Luan J., Willems S.M., Wu Y., Zhang X., Horikoshi M. (2021). The trans-ancestral genomic architecture of glycemic traits. Nat. Genet..

[66] Dupuis J., Langenberg C., Prokopenko I., Saxena R., Soranzo N., Jackson A.U., Wheeler E., Glazer N.L., Bouatia-Naji N., Gloyn A.L. (2010). New genetic loci implicated in fasting glucose homeostasis and their impact on type 2 diabetes risk. Nat. Genet..

[67] UK Biobank Neale Lab. http://www.nealelab.is/uk-biobank.

[68] Graham S.E., Clarke S.L., Wu K.-H.H., Kanoni S., Zajac G.J.M., Ramdas S., Surakka I., Ntalla I., Vedantam S., Winkler T.W. (2021). The power of genetic diversity in genome-wide association studies of lipids. Nature.

[69] Cedó L., Reddy S.T., Mato E., Blanco-Vaca F., Escolà-Gil J.C. (2019). HDL and LDL: potential new players in breast cancer development. J. Clin. Med..

[70] Dimou N.L., Papadimitriou N., Gill D., Christakoudi S., Murphy N., Gunter M.J., Travis R.C., Key T.J., Fortner R.T., Haycock P.C. (2019). Sex hormone binding globulin and risk of breast cancer: a Mendelian randomization study. Int. J. Epidemiol..

[71] Eketunde A.O. (2020). Diabetes as a risk factor for breast cancer. Cureus.

[72] Guo Y., Warren Andersen S., Shu X.-O., Michailidou K., Bolla M.K., Wang Q., Garcia-Closas M., Milne R.L., Schmidt M.K., Chang-Claude J. (2016). Genetically predicted body mass index and breast cancer risk: mendelian randomization analyses of data from 145,000 women of European descent. PLoS Med..

[73] Murphy N., Knuppel A., Papadimitriou N., Martin R.M., Tsilidis K.K., Smith-Byrne K., Fensom G., Perez-Cornago A., Travis R.C., Key T.J., Gunter M.J. (2020). Insulin-like growth factor-1, insulin-like growth factor-binding protein-3, and breast cancer risk: observational and Mendelian randomization analyses with ∼430 000 women. Ann. Oncol..

[74] Sun B.B., Maranville J.C., Peters J.E., Stacey D., Staley J.R., Blackshaw J., Burgess S., Jiang T., Paige E., Surendran P. (2018). Genomic atlas of the human plasma proteome. Nature.

[75] Bowden J., Del Greco M F., Minelli C., Davey Smith G., Sheehan N., Thompson J. (2017). A framework for the investigation of pleiotropy in two-sample summary data Mendelian randomization. Stat. Med..

[76] Davies N.M., Holmes M.V., Davey Smith G. (2018). Reading Mendelian randomisation studies: a guide, glossary, and checklist for clinicians. BMJ.

[77] Davey Smith G., Hemani G. (2014). Mendelian randomization: genetic anchors for causal inference in epidemiological studies. Hum. Mol. Genet..

[78] Sanderson E., Glymour M.M., Holmes M.V., Kang H., Morrison J., Munafò M.R., Palmer T., Schooling C.M., Wallace C., Zhao Q., Davey Smith G. (2022). Mendelian randomization. Nat. Rev. Methods Primers.

